# Algorithm for Determining Time Series of Phase Transformations in the Solid State Using Long-Short-Term Memory Neural Network

**DOI:** 10.3390/ma15113792

**Published:** 2022-05-26

**Authors:** Joanna Wróbel, Adam Kulawik

**Affiliations:** Department of Computer Science, Czestochowa University of Technology, Dabrowskiego 73, 42-201 Czestochowa, Poland; adam.kulawik@pcz.pl

**Keywords:** recurrent neural network, time series, phase transformation, CCT diagrams

## Abstract

In the numerical analysis of manufacturing processes of metal parts, many material properties depending on, for example, the temperature or stress state, must be taken into account. Often these data are dependent on the temperature changes over time. Strongly non-linear material property relationships are usually represented using diagrams. In numerical calculations, these diagrams are analyzed in order to take into account the coupling between the properties. An example of these types of material properties is the dependence of the kinetics of phase transformations in the solid state on the rate and history of temperature change. In literature, these data are visualized Continuous Heating Transformation (CHT) and Continuous Cooling Transformation (CCT) diagrams. Therefore, it can be concluded that time series analysis is important in numerical modeling. This analysis can also be performed using neural networks. This work presents a new approach to storing and analyzing the data contained in the discussed CCT diagrams. The application of Long-Short-Term Memory (LSTM) neural networks and their architecture to determine the correct values of phase fractions depending on the history of temperature change was analyzed. Moreover, an area of research was elements that determine what type of information should be stored by LSTM network coefficients, e.g., whether the network should store information about changes of single phase transformations, or whether it would be better to extract data from differences between several networks with similar architecture. The purpose of the studied network is strongly different from typical applications of artificial neural networks. The main goal of the network was to store information (even by overfitting the network) rather than some form of generalization that allows computation for unknown cases. Therefore, the authors primarily investigated in the ability of the layer-based LSTM network to store nonlinear time series data. The analyses presented in this paper are an extension of the issues presented in the paper entitled “Model of the Austenite Decomposition during Cooling of the Medium Carbon Steel Using LSTM Recurrent Neural Network”.

## 1. Introduction

The continuous development of knowledge in the area of technical fields places increasing demands on modern engineers. Currently, both during the design and implementation of manufacturing processes, special attention is paid to minimizing costs, reducing working time, and improving the efficiency of technological processes. Due to the use in the design of data contained in diagrams that bind the influence of various factors such as rate of change, temperature, strain, displacement, etc., it is necessary to use the error-free diagram analysis in models for autonomous optimization. Classical analysis based on the detection of intersections of lines (e.g., cooling lines) with the boundaries of the areas of phase transformation is both time-consuming and may not take into account all possible cases [1,2,3]. The certainty of taking into account all possibilities that may occur, for example, during optimization with the use of genetic algorithms (the occurrence of parameter combinations that are illogical from the user’s point of view) gives the possibility of using artificial intelligence tools for a mass search of solutions. For this reason, in this paper, the storage of nonlinear data in a multilayer network of the LSTM type is proposed.

Long-Short-Term Memory recurrent neural networks are capable of modeling tasks with multiple input variables [4,5]. This is a great advantage, especially in the case of time series prediction, where classical linear methods may be difficult to adapt, e.g., due to the amount of input data. LSTM is used in many very different fields of science and technology [6]. Lindemann et al. [7] applied LSTM networks to detect anomalies in the time series data. The detection is performed by evaluating the deviation of the actual system performance from the expected performance predicted by the network. On the other hand, Lee et al. [8] used two LSTM network models for real-time anomaly detection in their work. This also applies to the detection of anomalies in nonlinear real systems described in the work of [9]. Networks such as LSTM are often used for prediction in the field of machine control or repair. Nemani [10] used LSTM to predict the remaining useful life of bearings, providing better uncertainty estimates compared to traditional models.

Many theoretical and experimental papers on the application of LSTM in mechanics and heat treatment of metals have also been published. Choudhary et al. [11], in their paper, reviewed the literature on the application of deep learning in materials engineering. However, the work related to the application of LSTM is more about extracting information from textual data. In the method developed by Liu et al. [12], a convolutional neural network (CNN) and LSTM neural networks were combined. This hybrid approach allows the detection of defects in the weld pool. To enable multidimensional defect detection in the method developed by Liu et al. [13], CNN and LSTM were combined. The CNN model was used for image feature detection, while the LSTM was used to identify steel surface defects. Based on the results, it was found that the defect detection rate for the hybrid method was higher than that of the single CNN or LSTM method. Benabou [14] used the LSTM network in his paper to predict the stress in a brazed joint based on strain, strain rate, and temperature changes over time. The obtained results demonstrated good agreement with the training data obtained from the FEM model. On the other hand, Rabe et al. [15] compared the performance of deep learning methods—CNN, LSTM, and BiLSTM for detecting internal weld defects during the friction stir welding (FSW) process. The results demonstrate that CNNs are better suited for detecting internal voids than RNNs. Haghighi [16] used LSTM to predict path-dependent plasticity associated with material heterogeneity and anisotropy. The authors found that the LSTM-based model can capture J2 plasticity responses for both monotonic and arbitrary loading paths. Whereas Abueidda et al. [17] compared recurrent neural networks and a temporal convolutional network (TCN) to a periodic elastoplastic material, as well as to a more complex solidification model of thermo-viscoplastic steel.

Analyzing the above papers, it can be concluded that LSTM is used for prediction rather than knowledge storage. In the present paper, the authors focus on information storage via the LSTM network.

**Figure 1:**
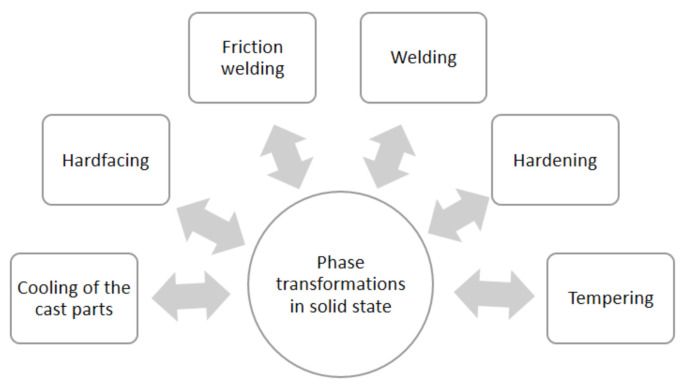
Examples of processes in which phase transformations in the solid state should be taken into account during modelling.

An important aspect of the analyzed processes are the phase transformations in the solid state. Phase transformations in the solid state are an important part of the modelling of many industrial processes (Figure 1) and taking them into account may have a strong influence on the accuracy of the obtained results. Consideration of phase transformations in the solid state is a necessary element that should be taken into account in the models of the stress state in the elasto-plastic range. These transformations can not only lead to improved properties of the heat-treated element but can also cause significant temporary stresses leading to the formation of cracks in the material. Important factors affecting the type of structures formed are the temperature and rate of heating and cooling, as well as the austenitizing temperature, among others. Taking into account the phase transformations can therefore lead to an optimization of the heat treatment process or the welding process of steel elements through an appropriate choice of the temperature change scheme [18,19,20]. Thus, diagrams that take these factors into account store strongly nonlinear data, which is a challenge for the users analyzing them or the algorithms using them.

LSTM networks are a fairly modern tool in the time series analysis; their previous applications are far from the scope of mechanical engineering or materials engineering. The data for the LSTM network were determined based on a macroscopic model of phase transformations in the solid state. Replacement of the model (in fact, quite simple equations) however, based on strongly nonlinear diagrams, allows one to quickly determine the phase transformations for one steel grade and one austenitizing temperature. An extension of the work, after correct verification of the functionality for one diagram, may be a generalization of the model for a specific group of steels. Storing information (network learning) for several similar grades in the authors’ understanding will allow for the proper functioning of such a model for chemical compositions’ intermediate to selected steel grades. Therefore, the presented paper focuses on demonstrating that it is possible to take the first step in this area and it is possible to replace the model and the diagram with the knowledge stored in the LSTM network. In addition, the level of difference between the results from both approaches will be so small that it will not affect the errors of the models using phase shares in the solid state.

The presented paper consists of two main parts:

- The first part deals with the description of the numerical model, which is replaced by a recurrent network and whose results represent learning, testing, and validation data. This section characterizes the analyzed variants of the input data structure and other parameters of the calculations.
- The second part deals with the presentation of the obtained results and the analysis of the data, as well as the conclusions regarding the proposed new approach.

## 2. Parameters and Methods

**Figure 2:**
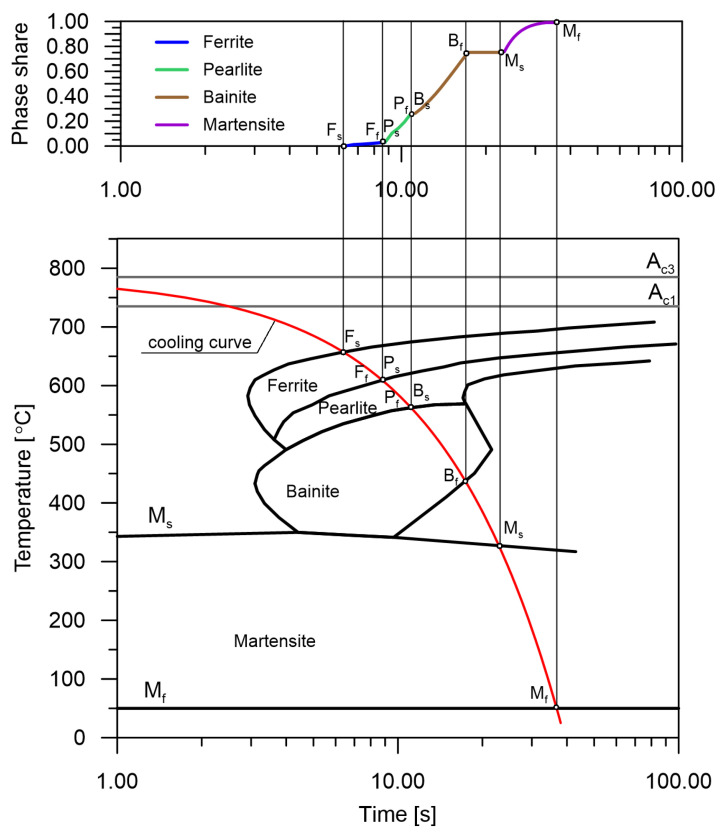
Analyzed diagram of phase transformations in the solid state [21].

Information from the CCT diagram was used as input to the RNN model (Figure 2). However, the analyzed CCT diagrams do not provide information on the behaviour of phase transformations between the start and end lines of the transformation. The use of line interpolation is not consistent with the actual kinetics of transformations. Therefore, the transition functions obtained from the Johnson–Mehl–Avrami–Kolmogorov (JMAK) macroscopic model of phase transformations in the solid state were used.

Many models for the determination of the kinetics of phase transformations in the solid state are presented in the literature, and they are characterized by different mathematical descriptions, as well as the degree of adaptation to the physical phenomena taking place during phase transformations. For the determination of the kinetics of diffusion transformations, the empirical Johnson–Mehl–Avrami–Kolmogorov equation [22] or, in the case of high heating rates, the modified Koistinen–Marburger equation [23] is most often used. On the other hand, the basic equation to determine the kinetics of martensite transformation is the Koistinen–Marburger equation.

To determine the increment of the austenitic phase, the JMAK equation of the form was used [24,25,26,27]

(1) $${\tilde{\eta }}_{A}\left(T,t\right)=1-exp\left(-b\left(T\right)t^{n(T)}\right)$$

where ${\tilde{\eta }}_{A}$ is the share of austenite formed in the heating process, the functions n(T) and b(T) are estimated according to the relation [21].

(2) $$\begin{array}{cc} n\left(T\right)=\frac{ln\left(\frac{ln(1-\eta_{f})}{ln(1-\eta_{s})}\right)}{ln\left(\frac{t_{f}\left(T\right)}{t_{s}\left(T\right)}\right)} & ,b\left(T\right)=\frac{-ln(1-\eta_{s})}{{\left(t_{s}\right)}^{n(T)}} \end{array}$$

where $t_{s}$ indicates the start time of the transformation, $t_{f}$ the end time of the transformation, $\eta_{s}$ and $\eta_{f}$ are the start and end shares of the forming phase. The start and end times of the phases were determined from the continuous kinetics model [28].

The values of phase fractions, i.e., ferrite, pearlite, and bainite, during the cooling process, are determined from the JMAK equation

(3) $$\eta_{\left(i\right)}\left(T,t\right)=\eta_{i}^{\%}\cdot \left({\tilde{\eta }}_{A}-\sum_{j\neq i}\eta_{j}\right)\left(1-exp\left(-b\left(T\right)t^{n(T)}\right)\right)$$

where $\eta_{i}$ is the share of the *i*-th phase created in the cooling process, and $\eta_{i}^{\%}$ is the final share of the phase (*i*) estimated from the CCT diagram.

The share of the martensite phase is calculated using the Koistinen–Marburger formula [23]

(4) $$\eta_{M}\left(T,t\right)=\left({\tilde{\eta }}_{A}-\sum_{i\neq M}\eta_{i}\right)\left(1-exp\left(-k{\left(M_{s}-T\right)}^{n}\right)\right)$$

where $M_{s}$ is the temperature of martensitic transformation start, *n* is an experimentally selected constant (for medium-carbon constructional steel n=1), while the value of the coefficient *k* is calculated from the relation

(5) $$k=-\frac{ln(1-\eta_{MAX})}{M_{s}-M_{f}}$$

where $\eta_{MAX}=0.99$ is the maximum share of martensite and $M_{f}$ is the finish temperature of martensitic transformation.

The accuracy of the model of the kinetics of phase transformations occurring during cooling is determined by the appropriate analysis of the CCT diagrams. From these diagrams, the start and end times of the phase transformations as well as their maximum possible shares are obtained. In this paper, a model that assumes that individual transformations follow one global kinetics is used.

In the presented model, it has been assumed that the macroscopic model of phase transformations in the solid state will be replaced by an RNN network. The choice of the network resulted from the fact that the austenite decomposition function is approximated as a time series. This model cooperates with the FEM model, which should take the phase transformations into account to determine the stress level. The presented network approximates the changes of transformations in time during the cooling process analogically to the classical analysis of CCT diagrams [21,26,28]. All calculations were performed in the software developed by the authors of the paper (phase transformation model in the solid state—sets for RNN). The numerical model, based on which the learning sets were determined, was verified experimentally based on dilatometric tests presented in the paper [25]. Keras library [29,30] was used for calculations of recurrent networks. The following computational parameters were assumed:

- The analysis starts when the $A_{c3}$ temperature exceeds 1058 °C (t = 0 s),
- The number of input data was equal to 3600 files,
- The set was divided proportionally into a 50% training set, 25% testing set, and 25% validation set. Data were assigned to each set randomly without repetition,
- Number of epochs: 2000, 4000, 10,000—due to the lack of progress on the issue of higher accuracy, after preliminary tests, the results obtained for 2000 and 4000 (taking into account the initial learning) epochs were selected for presentation,
- Batch size: 100,
- Number of LSTM layers: 3,
- Number of input sequence—100,
- The length of sequences complemented with zeros—83 (for example: for a speed of 12 K/s all elements of the sequence is non-zero and for a speed of 80 K/s 20 non-zero complemented with 63 zeros),
- One TimeDistributed layer,
- Number of iterations per 1 epoch—18,
- Cooling rate—from 10 to 80 K/s,
- Constant time step—0.5 s,
- Termination of the calculation of transformations after reaching austenite values below 1%.
- The learning process was carried out using Adam’s optimization method [31],
- The rectified linear unit activation function (ReLU) was used at the output of the network [32].
- To illustrate the changes and compare the models for the full range of speeds, the following control points were selected for analysis: point 1—cooling rate 10.4 K/s, point 2—cooling rate 27.7 K/s, point 3—cooling rate 45 K/s, point 4—cooling rate 62.3 K/s, and point 5—cooling rate 79.6 K/s.

**Figure 3:**
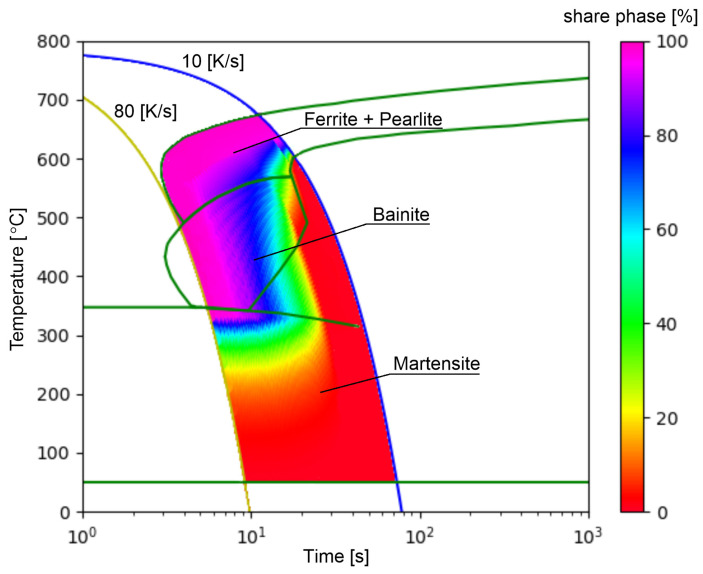
CTP diagram divided into analysed ranges—input/output data for RNN.

The selected range of cooling rates (Figure 3) is the most difficult area from the analyzed CCT diagram due to the strong nonlinearity of the transformations and the occurrence of all transformations in this area. The reference Figure 3 also shows the area of occurrence of only ferritic–pearlitic transformation (rates from 10–12.41 K/s). Despite the completely different transformation structure than in the rest of the area, the network model does not introduce large errors here, except for the point where the areas are connected. For this reason, the analysis of results for cooling rates below 12.41 K/s was limited.

**Figure 4:**
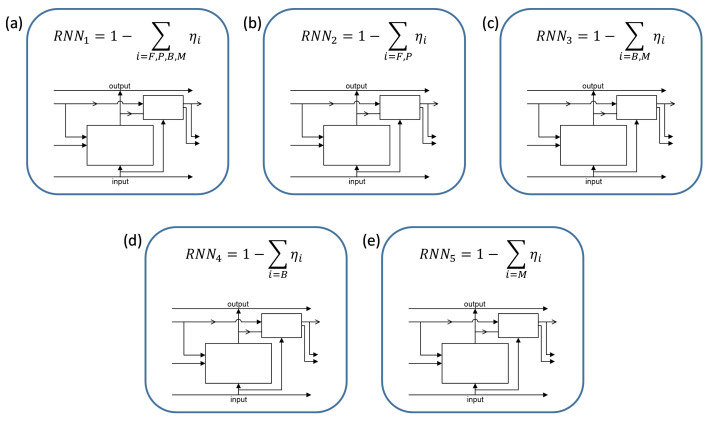
The considered configurations of data determined by RNN: (**a**) sum of all phases, (**b**) sum of ferrite and pearlite, (**c**) sum of bainite and martensite, (**d**) bainite, and (**e**) martensite.

Due to the large differences in the thermal and structural expansion of the individual phases [26], the results of the analysis cannot be the sum of the transformations but they must be given separately. It was determined based on tests that the results obtained for single phases from the presented model give a very large error, especially for cases without prelearning from the sum of phases. For this reason, cases of the extrapolation of data for individual phases from differences between different sums were analyzed (Table 1). Thus, a learning network was considered based on (Figure 4):

- Sum of all phases from the cooling process $RNN_{1}$ (ferrite, pearlite, bainite, martensite),
- Sum of diffusion phases $RRN_{2}$ (ferrite, pearlite),
- Sum of the quenching phases $RNN_{3}$ (bainite, martensite),
- Bainite content $RNN_{4}$,
- Martensite content $RNN_{5}$.

Based on the obtained results for the case of learning austenite distribution for the number of epochs equal to 100,000 it was determined that for a greater number of epochs the network error increases. This error does not mean that the network is overlearning because it is not able to generalize, but it means that it is not able to reproduce learning patterns with the same accuracy as for 4000 epochs. The error for 100,000 calculated by the formula (Equation (6)) had a value more than 68% higher than for 4000 epochs. The structure of underestimation/overestimation was similar for 4000 epochs.

**Figure 5:**
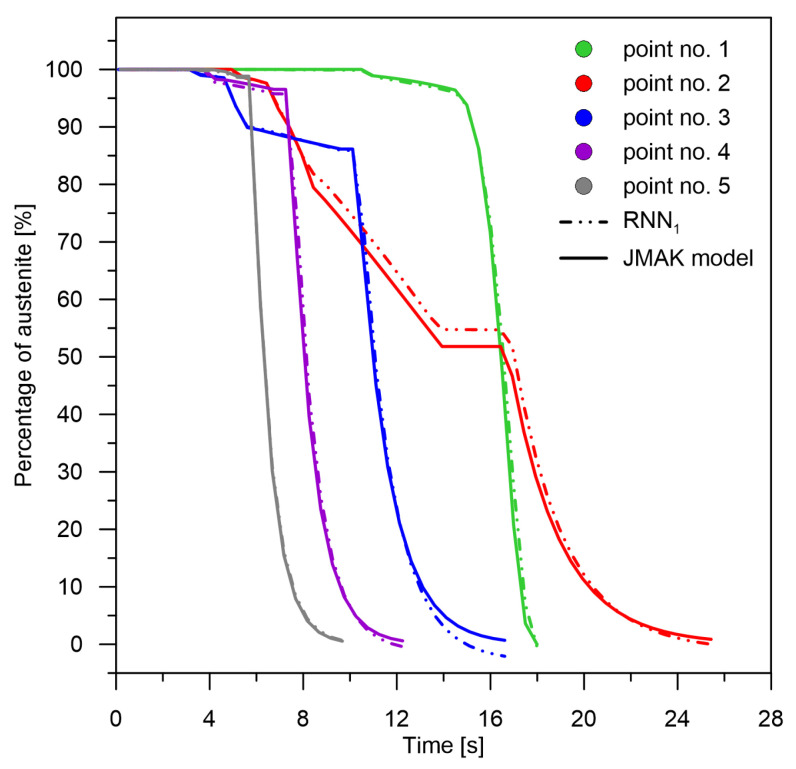
Kinetics of austenite transformation for selected points—consideration of weights in initial learning. Comparison of data calculated by the JMAK numerical model and data determined by the RNN.

It was considered to obtain diffusion transformations based on the difference between the sum of all phases and bainite and martensite calculated together or separately (Table 1). Martensite was obtained from the sum of bainite and martensite minus bainite, while bainite was obtained from the difference of the sum of bainite and martensite and martensite. All these values were determined from the model with pre-learning of weights for the sum of all phases (Figure 5). The determination of values for individual phases and their sums without considering pre-learning were also taken into account (Table 2).

Due to the fact that significantly better results were obtained when pre-learning was applied, it was assumed that the results obtained for the sum of all phases from the model presented in the authors’ previous work [28] could be used as pre-learning weights. The above remark means that the architecture of the network is consistent with the architecture in the mentioned work (Table 3).

## 3. Results and Conclusions

**Figure 6:**
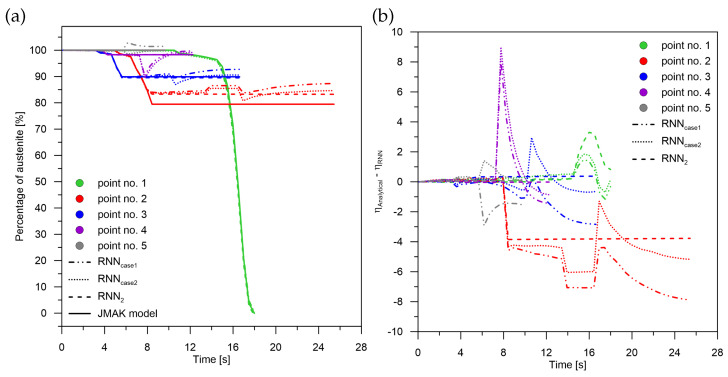
Phase transformation kinetics—case No. 1 and case No. 2. (**a**) Phase transformation $RNN_{2}$. (**b**) The difference between JMAK and RNN models.

Signal analysis for the ferrite–pearlite mixture from the three models based on the RNN network and the analytical model indicates significant discrepancies in the obtained results (Figure 6). Assuming that the individual models were based on the initial weights obtained from the austenite distribution (Figure 5), accuracy was obtained with relative error levels from −8 to 9. It can be noted that while the curve obtained from the model for the determination of a single phase (in this case ferrite and pearlite together), maintains the consistency of the derivative with the reference model (shape of the curve) and the difference is only in the level of the obtained value, then the results obtained from the difference of the network signals (austenite distribution minus bainite and martensite) indicate a strong influence of the remaining learning data from the ranges below the line of the end of the perlitic transformation. This error is cumulative for the deduction based on a single lattice as the sum of bainite+martensite. The difference in levels is particularly evident for the cooling rate range, where the maximum share of the ferritic–perlitic structure exceeds 20% (rates from 30 to 15 K/s), and at the same time the martensitic structure is formed. As could be predicted, the smallest error (−1 to 3 K/s) is for the signal where the transformation range is the largest (cooling velocities below 10.4 K/s). The absolute error increases significantly as the cooling rate increases.

**Figure 7:**
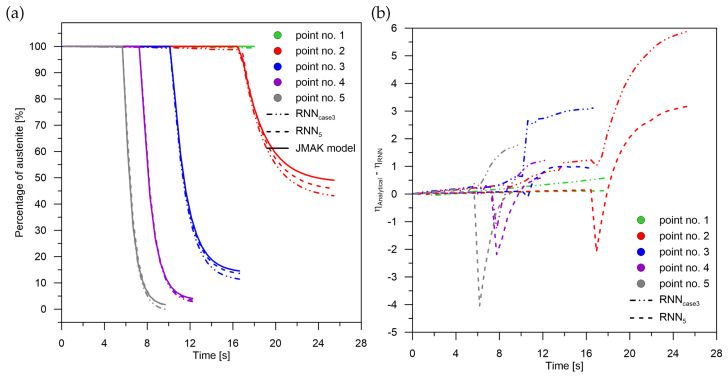
Phase transformation kinetics—case No. 3. (**a**) Phase transformation $RNN_{5}$. (**b**) The difference between JMAK and RNN models.

A similar trend in accuracy is observed in the martensitic transformation examples (Figure 7). The best level of accuracy is obtained from single-phase determination—the highest rates. With the use of the network signal difference for calculations in the case of martensite determination, the value of the relative error multiplied with a tendency of decreasing difference with decreasing cooling rate. The deviation of transformation curves obtained from RNN models increases with a decreasing magnitude of change. The error is significantly smaller for the curves with rapid changes. This can be observed especially in Figure 7 (compare rates in point No. 5 and point No. 2), Figure 9 (austenite distribution point No. 5 and point No. 2) and Figure 11b (increase in the sum of bainite and martensite—point No. 5 and point No. 2). Note that the figure shows relative errors. If we relate them to the size of the martensitic transformation, the errors for points No. 4 and No. 5 will decrease further.

**Figure 8:**
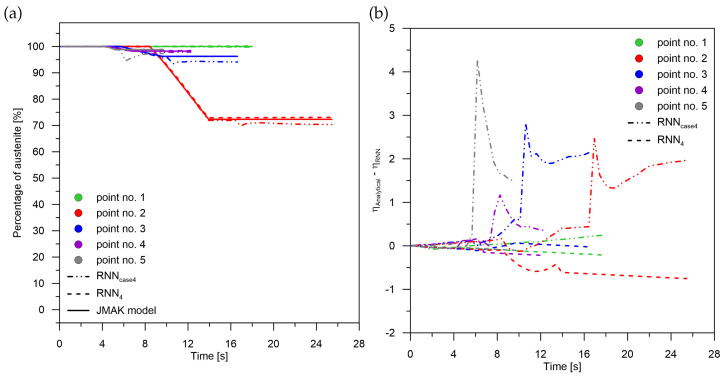
Phase transformation kinetics—case No. 4. (**a**) Phase transformation $RNN_{4}$. (**b**) The difference between JMAK and RNN models.

The error rates for the models describing the growth of the bainitic structure prove different relationships than for the diffusion transformations (Figure 8). The error level does not increase with decreasing rate (compare point No. 2 and point No. 3). On the error of the solution from the RNN model, the austenite → ferrite + pearlite transformation and the austenite → martensite transformation do not negatively affect the austenite → bainite transformation. The before and after signals are more of a corrective nature.

Modelling the transformations does not increase the error, as the final level of the transformation decreases. The curves for final shares at levels below 10% are not affected by greater inaccuracies (Figure 6 and Figure 8).

**Figure 9:**
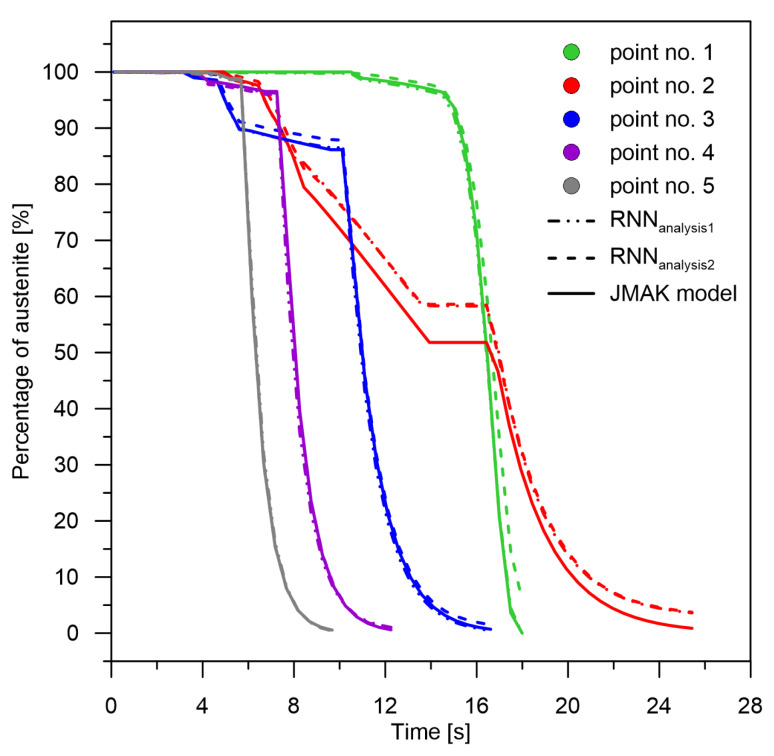
Phase transformation kinetics—analysis No. 1 and analysis No. 2.

During performing numerical experiments it was noticed that the attempt to store information by the LSTM network for single phases or pairwise sums leads to very high errors. It can be assumed that for these cases the RNN network does not learn. For this reason, it was decided to use initial weight values (Figure 5)—application of initial learning. The initial weight values for the separated signal models were taken from the sum-of-signals model (Figure 9).

**Figure 10:**
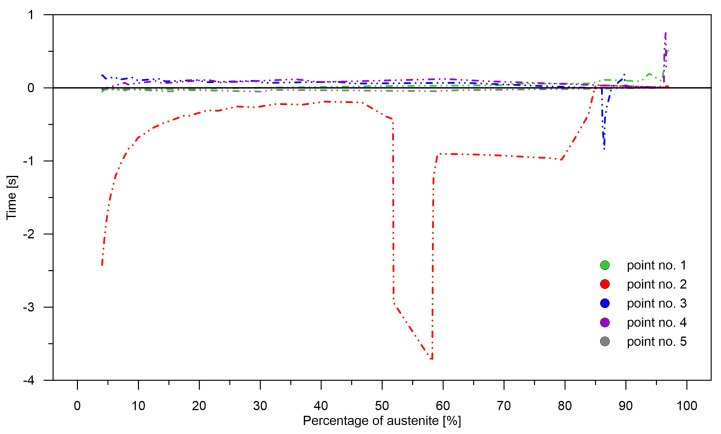
Analysis No. 1 and No. 2—rate of update of transformation kinetics.

One of the most important parameters is the rate of change adjustment over time Figure 10). It is understood as the size of the time interval needed to reach a given level of transformation with the occurrence of overestimation or underestimation. A small distance in time between appropriate transformation levels do not significantly affect the calculation, for example, the stress level in the heat-treated element. However, a large difference between adjacent points and the accumulation of underestimations or overestimations can result in the appearance of significant temporary stresses.

**Figure 11:**
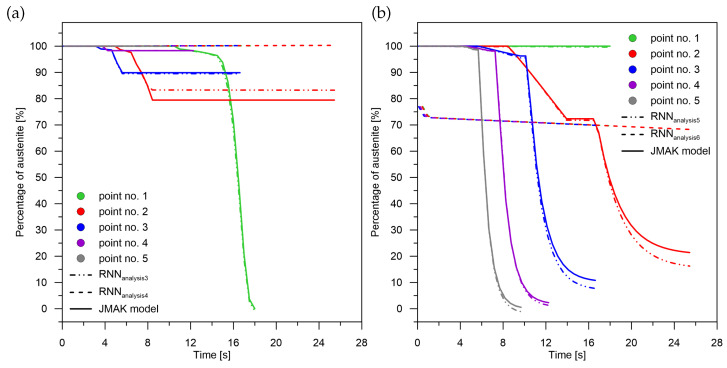
Analysis of phase transformation kinetics. (**a**) Analysis No. 3 and No. 4. (**b**) Analysis No. 5 and No. 6.

**Figure 12:**
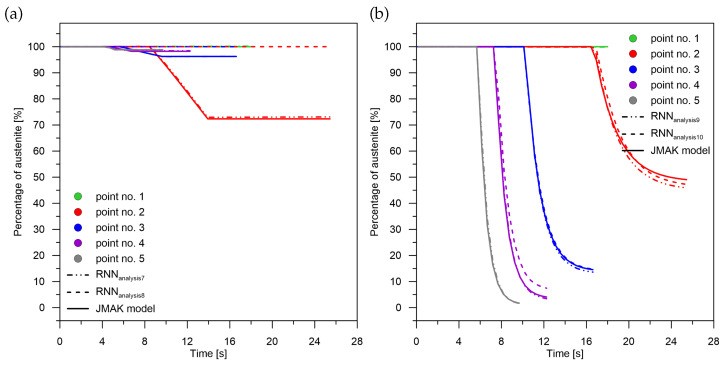
Analysis of phase transformation kinetics. (**a**) Analysis No. 7 and No. 8. (**b**) Analysis No. 9 and No. 10.

Applying pre-learning at the signal sum level for recurrent networks results in:

- For diffusion transformations (Figure 11a) to obtain correct values with a maximum absolute error of no more than 1% for the middle part of the interval (point No. 3). The error decreases as the cooling rate value increases. It can be observed that when there is no martensitic transformation (point No. 1) using the network with pre-learning results in an error of only 1.03% for a transformation in the range of 99%. The largest error of 3.7% can be observed when there is a significant amount of ferritic–perlitic transformation and martensitic transformation (point No. 2–27.7 K/s);
- For quenching transformations (Figure 11b), increasing the cooling rate results in a decrease in both the relative error (from 5 to 1%) with an absolute error of no more than 0.1%. The error characteristics are indicative of the overestimation of the transformation by the RNN,
- In the range of bainitic transformation (Figure 12a), no significant changes in error values were observed with changes in cooling rate,
- In terms of martensitic transformation (Figure 12b), it cannot be concluded that the use of pre-learning in every case is better than the use of networks without pre-learning. However, on average, it can be demonstrated that the error is higher for the network without pre-learning.

Failure to apply pre-learning at the sum-of-signals level for recurrent networks results in:

- Correctly calculating the value for the last in-time component of the signal (martensite) (Figure 12b),
- Lack of correct results for the initial and middle components of the signal both for their sum and for their complete separation (Figure 11 and Figure 12a).

**Figure 13:**
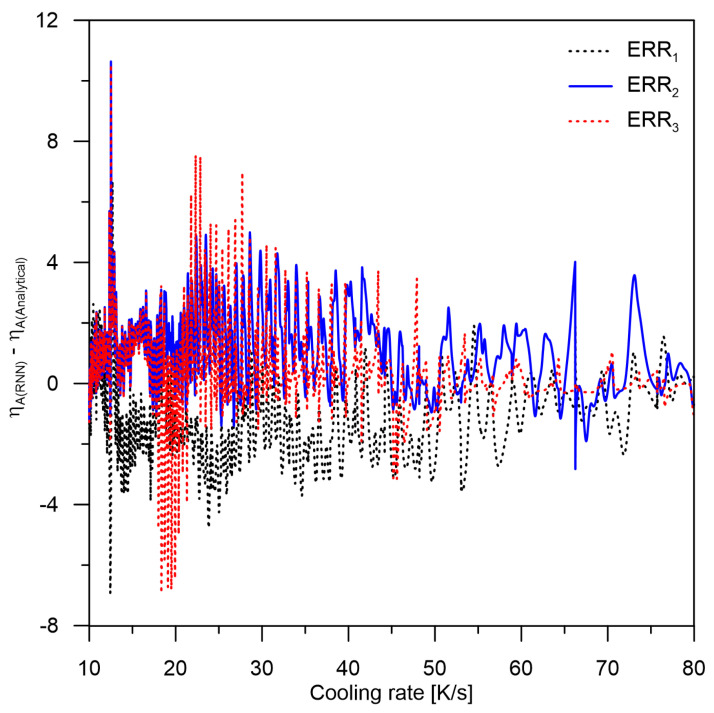
Visualization of the difference $\eta_{A(RNN)}-\eta_{A(Analytical)}$ for a cooling rate range of 10–80 K/s.

The analytical model demonstrates that the maximum level of the residual austenite after the cooling process is no more than 1%. This assumption is due to the adopted parameters of the Equation (2) ($\eta_{s}$ = 0.01, $\eta_{f}$ = 0.99). The error rate from the RNN model after the cooling process gives information on how much of the transformation was missed. These data are important and characterize the error rate of the network without taking into account the delays or accelerations of the phase transformations that are visible in Figure 13 and Table 4.

(6) $$ERR_{int}=\int_{10}^{80}\left|\eta_{A(RNN)}-\eta_{A(Analytical)}\right|dCr$$

where: $\eta_{A(RNN)}$—austenite share determined from RNN, $\eta_{A(Analytical)}$—austenite share from FEM model, and $d_{Cr}$—cooling rate [K/s].

- $ERR_{1}$—the difference between values calculated from RNN and values from the analytical model without consideration of preweights after the cooling process (2000 epochs),
- $ERR_{2}$—the difference between the values calculated from the RNN and the values from the analytical model with preweights included (4000 epochs),
- $ERR_{3}$—the difference between the values calculated from the RNN and the values from the analytical model with preweights included for a temperature of 350 °C.

Since the average error rate (unprocessed austenite level) from the RNN is not higher than 1.25 (Equation (6)), it can be assumed that this error rate is moderately small. However, it can locally reach up to more than 10 %. Thus, it is not important whether the austenite has been completely transformed (no error in this range) but whether the kinetics of the transformation proceeded according to the analytical model. After analyzing the results, it can be indicated that the largest error will be generated by the occurrence of delays or accelerations occurring locally in time.

The level of correlation between the values of the pre-learning weights (errors for the pre-learning network) and the results obtained from learning for the cases: case No. 1—case No. 4 and analysis No. 1—analysis No. 10 was checked (Figure 6, Figure 7, Figure 8, Figure 9, Figure 10, Figure 11 and Figure 12)—no correlation was found between the pre-learning and proper learning errors.

When analyzing the maximum values of errors, it can be concluded that appearing local maxima and minima influence the level of errors in the nearest neighbours due to the cooling velocity. The possibility of using the average of transformations for several close cooling speeds should be considered. It should be noted, however, that averaging the results in this way may lead to an increase in the error level if the difference in rates is significant. The authors will analyze this case in upcoming papers.

Due to its structure, the LSTM network can accurately determine the phase composition values not only at the end of the transformation but also during the cooling process even for the changing values of rate. The presented results suggest that the discussed LSTM model effectively replaces the model based on analytical equations. The levels of differences between the analytical model and the RNN model for neighbouring rates should not cause a high level of errors in practical applications of the discussed model, e.g., in stress analysis of heat-treated steel. Such a promising level of accuracy makes it possible to suppose that the approach described in the paper can be generalized for a model of phase transformations for a specific group of steels or one steel grade with different austenitizing temperatures. This will be further investigated by the authors.

## Figures and Tables

**Table 1:** Analyzed configurations of the considered examples.

Case No.	Operations
1	$RNN_{1}-RNN_{3}\Rightarrow RNN_{2}$
2	$RNN_{1}-\left(RNN_{4}+RNN_{5}\right)\Rightarrow RNN_{2}$
3	$RNN_{3}-RNN_{4}\Rightarrow RNN_{5}$
4	$RNN_{3}-RNN_{5}\Rightarrow RNN_{4}$

**Table 2:** Analyzed configurations of learning data and pre-learning.

	Analysis No.									
	1	2	3	4	5	6	7	8	9	10
Number of RNN network	$RNN_{1}$		$RNN_{2}$		$RNN_{3}$		$RNN_{4}$		$RNN_{5}$	
Initial weight	1	0	1	0	1	0	1	0	1	0

**Table 3:** Summary of model parameters.

Layer (Type)	Output Shape	Param
lstm_1 (LSTM)	(100, 83, 83)	28,220
lstm_2 (LSTM)	(100, 83, 83)	55,444
lstm_3 (LSTM)	(100, 83, 83)	55,444
time_distributed_1 (TimeDist)	(100, 83, 1)	84
activation_1 (Activation)	(100, 83, 1)	0
Total params: 139,192		
Trainable params: 139,192		
Non-trainable params: 0		

**Table 4:** Summary of error values (residual austenite) according to Equation (6).

	${\mathrm{ERR}}_{1}$	${\mathrm{ERR}}_{2}$	${\mathrm{ERR}}_{3}$
Above baseline (y = 0) area	7.34	77.73	32.99
Below baseline (y = 0) area	89.27	8.05	15.50
Total area	96.61	85.79	48.49

## Data Availability

The data presented in this study are available at https://icis.pcz.pl/~jwrobel/MDPI_data.zip (accessed on 2 May 2022).
